# Efficacy of glutathione for the treatment of nonalcoholic fatty liver disease: an open-label, single-arm, multicenter, pilot study

**DOI:** 10.1186/s12876-017-0652-3

**Published:** 2017-08-08

**Authors:** Yasushi Honda, Takaomi Kessoku, Yoshio Sumida, Takashi Kobayashi, Takayuki Kato, Yuji Ogawa, Wataru Tomeno, Kento Imajo, Koji Fujita, Masato Yoneda, Koshi Kataoka, Masataka Taguri, Takeharu Yamanaka, Yuya Seko, Saiyu Tanaka, Satoru Saito, Masafumi Ono, Satoshi Oeda, Yuichiro Eguchi, Wataru Aoi, Kenji Sato, Yoshito Itoh, Atsushi Nakajima

**Affiliations:** 1 Department of Gastroenterology and Hepatology, Yokohama City University Graduate School of Medicine, Yokohama, Japan; 2 Division of Hepatology and Pancreatology, Department of Internal Medicine, Aichi Medical University, Aichi, Japan; 3 Department of Biostatistics, Yokohama City University Graduate School of Medicine, Yokohama, Japan; 4 Department of Gastroenterology and Hepatology, Kyoto Prefectural University of Medicine, Kyoto, Japan; 5 Center for Digestive and Liver Diseases, Nara City Hospital, Nara, Japan; 6 Department of Gastroenterology and Hepatology, Kochi Medical School, Kochi, Japan; 7 Liver Center, Saga University Hospital, Saga, Japan; 8 Division of Applied Life Sciences, Graduate School of Life and Environmental Sciences, Kyoto Prefectural University, Kyoto, Japan; 9 Division of Applied Biosciences, Graduate School of Agriculture, Kyoto University, Kyoto, Japan

**Keywords:** Nonalcoholic fatty liver disease, Glutathione, Controlled attenuation parameter

## Abstract

**Background:**

Glutathione plays crucial roles in the detoxification and antioxidant systems of cells and has been used to treat acute poisoning and chronic liver diseases by intravenous injection. This is a first study examining the therapeutic effects of oral administration of glutathione in patients with nonalcoholic fatty liver disease (NAFLD).

**Methods:**

The study was an open label, single arm, multicenter, pilot trial. Thirty-four NAFLD patients diagnosed using ultrasonography were prospectively evaluated. All patients first underwent intervention to improve their lifestyle habits (diet and exercise) for 3 months, followed by treatment with glutathione (300 mg/day) for 4 months. We evaluated their clinical parameters before and after glutathione treatment. We also quantified liver fat and fibrosis using vibration-controlled transient elastography. The primary outcome of the study was the change in alanine aminotransferase (ALT) levels.

**Results:**

Twenty-nine patients finished the protocol. ALT levels significantly decreased following treatment with glutathione for 4 months. In addition, triglycerides, non-esterified fatty acids, and ferritin levels also decreased with glutathione treatment. Following dichotomization of ALT responders based on a median 12.9% decrease from baseline, we found that ALT responders were younger in age and did not have severe diabetes compared with ALT non-responders. The controlled attenuation parameter also decreased in ALT responders.

**Conclusions:**

This pilot study demonstrates the potential therapeutic effects of oral administration of glutathione in practical dose for patients with NAFLD. Large-scale clinical trials are needed to verify its efficacy.

**Trial registration:**

UMIN000011118 (date of registration: July 4, 2013).

## Background

Nonalcoholic fatty liver disease (NAFLD) is an important cause of chronic liver injury worldwide [1, 2]. The spectrum of NAFLD ranges from nonalcoholic fatty liver to nonalcoholic steatohepatitis (NASH), cirrhosis, and hepatocellular carcinoma [3]. NAFLD is associated with metabolic syndromes and the incidence of NAFLD has increased over time [4, 5]. First-line treatment for NAFLD is lifestyle modification to achieve weight reduction, particularly through diet and exercise [6]. However, weight reduction is very difficult to accomplish and maintain. Effective therapy for NAFLD has not yet been established.

Glutathione, γ-L-glutamyl-L-cysteinyl-glycine, is a tripeptide present in every cell of the human body [7]. Although its functions are complex and remain the subject of current research, glutathione is thought to play crucial roles in the detoxification and antioxidant systems in cells. Because a reduction of glutathione levels in cells has been found to increase the risks for diseases and poisoning, direct intravenous injection of glutathione has been used to treat patients with chronic liver diseases and poisoning [8, 9].

Glutathione is synthesized in cells from glutamic acid, cysteine, and glycine. Cysteine and glycine are generated from methionine and serine, respectively, and glutamic acid is synthesized from α-ketoglutarate, a metabolite of glucose. These amino acids are generally supplied from food. It has been reported that oral administration of glutathione did not change the levels of glutathione and glutathione disulfide in the deproteinized fraction of blood [10], and it has been suggested that orally administered glutathione is degraded into constituting amino acids and does not exert specific activity beyond the amino acid source. However, it has been reported that glutathione can pass through the mono layer of Caco-2 cells without degradation [11]. In addition, Park et al. reported an increase in the protein-bound form of glutathione in human blood after oral administration, while glutathione in the deproteinized fraction did not change [12]. These studies suggest that orally administered glutathione is absorbed into the blood and might have effects on the redox status in the human body. Such findings have encouraged us to examine the therapeutic effects of oral administration of glutathione on NAFLD.

The objective of the current study was to demonstrate the therapeutic potential of oral administration of glutathione in an open-label, single-arm, multicenter, pilot study prior to subsequent large-scale clinical trials. In this study, we compared clinical parameters before and after treatment with glutathione. We also evaluated controlled attenuation parameter (CAP) and liver stiffness measurement (LSM), as determined by vibration-controlled transient elastography (VCTE).

## Methods

### Patients and study design

The study protocol was conducted in accordance with the guidelines contained within the Declaration of Helsinki and was approved by the ethics committees of Yokohama City University and Kyoto Prefectural University of Medicine. Written informed consent was obtained from all participants before entry into the study. The trial is registered with the University Hospital Medical Information Network (UMIN) Clinical Trials Registry (UMIN000011118).

Patient enrollment began in January 2014 and ended when the target sample size was reached in September 2014. Follow-up of participants ended in December 2014. We prospectively evaluated 34 NAFLD patients with liver dysfunction. NAFLD was diagnosed based on ultrasonography. All 34 patients provided a detailed medical history and underwent a physical examination. Patients were excluded if they had infectious hepatitis (hepatitis B or C or Epstein–Barr virus infection), autoimmune hepatitis, primary biliary cirrhosis, sclerosing cholangitis, hemochromatosis, α1-antitrypsin deficiency, Wilson’s disease, drug-induced hepatitis, alcoholic hepatitis, or excessive alcohol consumption (present or past consumption of >20 g alcohol/day). No NAFLD patient had clinical evidence of hepatic decompensation, such as hepatic encephalopathy, ascites, variceal bleeding, or a serum bilirubin level greater than twice the upper limit of normal. All patients were started on a standard diet (30 kcal/kg/day, consisting of 50–60% carbohydrate, 20–30% fat, and 15–20% protein) and received exercise counseling beginning 3 months before glutathione treatment. Exercise consisted of 5–6 metabolic equivalents for 30 min daily. Patients taking medication for lifestyle-related comorbid diseases, such as hypertension, dyslipidemia, and diabetes, were included; however, no change in medication or dose was allowed.

Because serum alanine aminotransferase (ALT) levels have been reported to predict the histological course of NASH and because strict control of ALT is required to prevent the progression of NASH [13], the primary outcome of this study was a change in ALT levels.

### Anthropometric and laboratory evaluations

Patient weight and height were measured using a calibrated scale after patients removed their shoes and any heavy clothing. Venous blood samples were obtained after patients had fasted overnight (12 h). Platelet counts and concentrations of fasting blood sugar (FBS), hemoglobin A1c (HbA1c), immunoreactive insulin (IRI), high-density lipoprotein (HDL) cholesterol, low-density lipoprotein (LDL) cholesterol, triglycerides, non-esterified fatty acids (NEFA), aspartate aminotransferase (AST), ALT, γ-glutamyl transpeptidase, ferritin, and type IV collagen 7 were measured using standard laboratory techniques before and after glutathione treatment. Patients with FBS ≥126 mg/dL, HbA1c ≥6.5%, and/or currently using antidiabetic medication were defined as having diabetes according to the criteria of the Japan Diabetes Society [14].

Glutathione in the deproteinized fraction and protein-bound fraction of plasma were determined using the method described by Park et al. [12]. Briefly, 100 μL of plasma was mixed with three parts ethanol. The supernatant was used as the deproteinized fraction. The precipitate was extracted using 100 μL of 5% trichloroacetic containing 2% 2-mercaptoethanol. The supernatant was used as the protein-bound fraction. Glutathione in these fractions were alkalized and derivatized with 6-aminoquinolyl-N hydroxy succinimidyl carbamate as described previously. The derivatives were resolved and detected using liquid chromatography/electron spray ionization/tandem mass spectrometry in multi-reaction monitoring mode.

### Vibration-controlled transient elastography

VCTE was performed using an M-probe device (Fibroscan; EchoSens, Paris, France). Details of the technique and the examination procedure for LSM have been described previously [15, 16]. CAP was measured using VCTE to stage steatosis. The technique is a proprietary algorithm based on the ultrasonic attenuation coefficient of the shear wave of VCTE, an estimate of the total ultrasonic attenuation at 3.5 MHz. CAP uses the same radiofrequency data as LSM and is only appraised if the acquisition is valid. It is expressed in decibels per meter. Measurements were obtained from the right lobe of the liver through the intercostal spaces, with a patient lying in the dorsal decubitus position and the right arm in maximal abduction. Only VCTE measurements based on at least 10 valid shots and success rates ≥60% were considered reliable and were used for statistical analysis.

### Statistical analysis

Data are expressed as mean ± standard deviation, unless indicated otherwise. The sample size was determined by reference to a previous report [17]. We estimated that with this sample size, the study would have 80% power to detect an absolute difference in the rate of improvement in ALT of 30 percentage points, with a two-sided type 1 error of 0.05. All statistical analyses were performed using JMP ver. 11.2.0 software (SAS Institute, Cary, NC, USA). Univariate comparisons between patient groups were analyzed using the Student’s *t*-test or the Mann–Whitney’s U-test, as appropriate. A *p-*value <0.05 was considered statistically significant.

## Results

### Biochemical response after 4 months of glutathione treatment

**Fig. 1:**
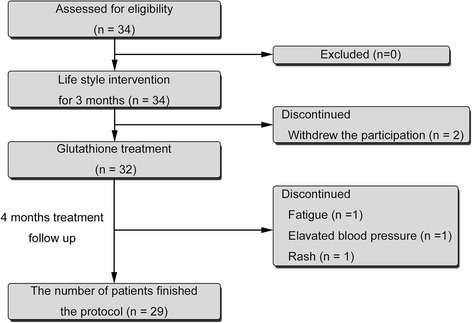
Study flow chart showing patient allocation

The study flowchart is shown in Fig. 1. Of the 34 patients enrolled, two withdrew before the start of treatment. The remaining 32 were treated with L-glutathione (300 mg/day; KOHJIN Life Sciences, Tokyo, Japan, US FDA GRAS #GRN000293) for 4 months by oral administration. Twenty-nine patients (14 men, 15 women, mean age 56.0 ± 13.3 years) finished the study protocol. Three patients dropped out, one each owing to fatigue, elevated blood pressure, and rash.

The clinical and laboratory characteristics of the study participants are shown in Table 1. Twenty-four patients (82.8%) had dyslipidemia and 12 (41.4%) were taking statins. Fourteen patients (48.3%) had diabetes.

**Table 1:** Characteristics of patients before and after glutathione treatment (*n* = 29)

	Before treatment	After treatment	*P*-value
Age (year)	56.0 ± 13.3		-
Male/female (n)	14/15		-
Dyslipidemia (%)	24 (82.8)		-
Statin (%)	12 (41.4)		-
Diabetes (%)	14 (48.3)		-
BMI (kg/m^2^)	26.5 ± 3.9	26.5 ± 3.9	0.32
FBS (mg/dL)	118.4 ± 34.3	120.0 ± 27.8	0.24
IRI (μU/mL)	23.1 ± 29.8	23.4 ± 33.8	0.38
HbA_1c_ (%)	6.37 ± 1.18	6.46 ± 1.23	0.016
HDL cholesterol (mg/dL)	55.2 ± 16.3	55.0 ± 15.4	0.32
LDL cholesterol (mg/dL)	114.0 ± 28.8	111.3 ± 28.0	0.08
Triglycerides (mg/dL)	195.2 ± 135.9	163.6 ± 121.9	0.007
NEFA (μEq/L)	651.2 ± 242.5	533.5 ± 209.7	0.013
AST (IU/L)	46.7 ± 17.2	47.6 ± 21.2	0.39
ALT (IU/L)	68.9 ± 36.1	58.1 ± 33.5	0.014
GGT (IU/L)	70.4 ± 46.5	66.6 ± 47.5	0.29
Ferritin (ng/mL)	219.8 ± 150.8	194.4 ± 139.2	0.015
Platelet count (×10^4^ /μL)	20.8 ± 5.7	20.9 ± 5.3	0.30
Type IV collagen 7 s	5.08 ± 1.95	4.84 ± 1.34	0.40
Glutathione in protein fraction (μM)	1.42 ± 0.87	0.93 ± 0.63	0.010
Glutathione in deproteinized fraction (μM)	0.025 ± 0.040	0.019 ± 0.024	0.27
CAP (db/m)	295.7 ± 44.9	285.4 ± 48.8	0.07
LSM (kPa)	9.94 ± 4.93	9.24 ± 4.48	0.16

Data are expressed as mean ± standard deviation. *BMI* body mass index, *FBS* fasting blood sugar, *IRI* immunoreactive insulin, *HBA1c*, hemoglobin A1c, *HDL cholesterol* high-density lipoprotein cholesterol, *LDL cholesterol* low-density lipoprotein cholesterol, *NEFA* non-esterified fatty acid, *AST* aspartate aminotransferase, *ALT* alanine aminotransferase, *GGT* γ-glutamyl transpeptidase, *CAP* controlled attenuation parameter, *LSM* liver stiffness measurement

After 4 months of glutathione treatment, ALT levels decreased significantly. Glutathione treatment decreased the concentrations of triglycerides, NEFA, and ferritin. HbA1c levels increased after glutathione treatment. Unexpectedly, glutathione in the plasma protein fraction decreased significantly after glutathione treatment. There was no significant difference in glutathione levels in the deproteinized fraction. Although glutathione treatment did not significantly affect CAP and LSM values, both tended to decrease.

### Glutathione treatment improved CAP values in ALT responders

The median decrease in ALT level from baseline was 12.9%. The 29 patients were divided into ALT responders (*n* = 15), defined as those with an ALT reduction ≥12.9%, and ALT non-responders (*n* = 14), defined as those with an ALT reduction <12.9%, and the factors associated with responses to glutathione were evaluated (Table 2). ALT responders were significantly younger than ALT non-responders (50.7 ± 12.1 years vs. 61.7 ± 12.4 years, *p* = 0.011). Body mass index (BMI) did not differ between ALT responders and non-responders (26.5 ± 4.1 kg/m^2^ vs. 26.6 ± 3.8 kg/m^2^, *p* = 0.47). Although the percentages of ALT responders and non-responders with dyslipidemia did not differ (80.0% vs. 85.7%, *p* = 0.68), HDL cholesterol and LDL cholesterol levels were higher in ALT responders. Rates of statin use tended to be lower in ALT responders than in non-responders (26.7% vs. 57.1%, *p* = 0.10). Rates of diabetes also tended to be lower (33.3% vs. 64.3%, *p* = 0.10) and HbA1c levels were significantly lower in ALT responders compared with non-responders. There were no significant differences in glutathione levels in the plasma protein and deproteinized fractions between ALT responders and non-responders before glutathione treatment.

**Table 2:** Characteristics of ALT responders and non-responders

	ALT responders (*n* = 15)	ALT non-responders (*n* = 14)	*P*-value
Age (year)	50.7 ± 12.1	61.7 ± 12.4	0.011
Male/female (n)	9 (6)	5 (9)	0.191
Dyslipidemia (%)	12 (80.0)	12 (85.7)	0.68
Statin (%)	4 (26.7)	8 (57.1)	0.10
Diabetes (%)	5 (33.3)	9 (64.3)	0.10
BMI (kg/m^2^)	26.5 ± 4.1	26.6 ± 3.8	0.47
FBS (mg/dL)	115.7 ± 37.9	121.4 ± 31.2	0.33
IRI (μU/mL)	23.5 ± 22.7	22.6 ± 38.0	0.47
HbA_1c_ (%)	5.94 ± 1.03	6.9 ± 1.2	0.019
HDL cholesterol (mg/dL)	60.3 ± 18.6	49.7 ± 11.7	0.04
LDL cholesterol (mg/dL)	124.5 ± 33.1	102.9 ± 18.6	0.021
Triglycerides (mg/dL)	202.4 ± 164.1	187.6 ± 103.3	0.39
NEFA (μEq/L)	720.2 ± 285.0	563.3 ± 143.0	0.055
AST (IU/L)	46.3 ± 20.2	47.1 ± 14.1	0.45
ALT (IU/L)	77.1 ± 38.6	60.1 ± 32.2	0.104
GGT (IU/L)	81.9 ± 58.0	58.0 ± 26.6	0.085
Ferritin (ng/mL)	260.2 ± 164.5	176.6 ± 126.1	0.07
Platelet count (×10^4^ /μL)	20.3 ± 4.6	21.3 ± 6.8	0.32
Type IV collagen 7 s	4.61 ± 1.13	5.59 ± 2.51	0.09
Glutathione in protein fraction (μM)	1.53 ± 0.92	1.27 ± 0.83	0.230
Glutathione in deprotenized fraction (μM)	0.017 ± 0.020	0.036 ± 0.057	0.116
CAP (db/m)	300.3 ± 41.1	290.4 ± 50.2	0.29
LSM (kPa)	8.71 ± 4.63	11.36 ± 5.05	0.080

Data are expressed as mean ± standard deviation

*Abbreviations: BMI* body mass index, *FBS* fasting blood sugar, *IRI* immunoreactive insulin, *HBA1c* hemoglobin A1c, *HDL cholesterol* high density lipoprotein cholesterol, *LDL cholesterol* low density lipoprotein cholesterol, *NEFA* non-esterified fatty acid, *AST* asparate aminotransferase, *ALT* alanine aminotransferase, *GGT* γ-glutamyl transpeptidase, *CAP* controlled attenuation parameter, *LSM* liver stiffness measurement

**Fig. 2:**
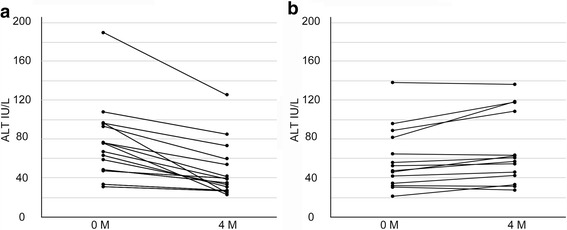
Alanine aminotransferase (ALT) levels before and after treatment with glutathione in **a** ALT responders and **b** ALT non-responders

The characteristics of ALT responders and non-responders before and after glutathione treatment are shown in Table 3. Glutathione treatment decreased ALT levels in ALT responders (Fig. 2a) but increased AST and ALT levels in ALT non-responders (Fig. 2b). In ALT responders, glutathione treatment decreased NEFA, ferritin, and HDL cholesterol levels but increased HbA1c levels. In ALT non-responders, glutathione treatment reduced triglyceride levels but increased FBS levels. Glutathione treatment significantly decreased glutathione in the plasma protein-boud fraction in ALT responders; there was no change in ALT non-responders. Surprisingly, CAP values were significantly reduced in ALT responders; there were no differences in ALT non-responders.

**Table 3:** Characteristics of ALT responders and non-responders before and after glutathione treatment

	ALT responders (*n* = 15)			ALT non-responders (*n* = 14)		
	Before treatment	After treatment	*P*-value	Before treatment	After treatment	*P*-value
Age (year)	50.7 ± 12.1		-	61.7 ± 12.4		-
Male/female (n)	9 (6)		-	5 (9)		-
Dyslipidemia (%)	12 (80.0)		-	12 (85.7)		-
Statin (%)	4 (26.7)		-	8 (57.1)		-
Diabetes (%)	5 (33.3)		-	9 (64.3)		-
BMI (kg/m^2^)	26.5 ± 4.1	26.5 ± 4.0	0.23	26.6 ± 3.8	26.4 ± 3.9	0.45
FBS (mg/dL)	115.7 ± 37.9	113.0 ± 23.0	0.38	121.4 ± 31.2	128.2 ± 31.4	0.004
IRI (μU/mL)	23.5 ± 22.7	17.9 ± 14.3	0.14	22.6 ± 38.0	30.5 ± 31.4	0.11
HbA_1c_ (%)	5.94 ± 1.03	6.08 ± 1.10	0.017	6.9 ± 1.2	6.89 ± 1.28	0.14
HDL cholesterol (mg/dL)	60.3 ± 18.6	57.4 ± 16.7	0.001	49.7 ± 11.7	52.2 ± 13.8	0.08
LDL cholesterol (mg/dL)	124.5 ± 33.1	117.1 ± 34.8	0.06	102.9 ± 18.6	104.1 ± 14.5	0.41
Triglycerides (mg/dL)	202.4 ± 164.1	178.8 ± 157.6	0.15	187.6 ± 103.3	146.2 ± 62.1	0.003
NEFA (μEq/L)	720.2 ± 285.0	576.0 ± 230.1	0.032	563.3 ± 143.0	473.9 ± 170.7	0.13
AST (IU/L)	46.3 ± 20.2	40.0 ± 20.7	0.11	47.1 ± 14.1	55.8 ± 19.3	0.003
ALT (IU/L)	77.1 ± 38.6	47.9 ± 28.1	<0.0001	60.1 ± 32.2	68.9 ± 36.3	0.005
GGT (IU/L)	81.9 ± 58.0	64.8 ± 48.4	0.07	58.0 ± 26.6	68.5 ± 48.3	0.06
Ferritin (ng/mL)	260.2 ± 164.5	217.7 ± 162.3	0.015	176.6 ± 126.1	169.5 ± 109.9	0.28
Platelet count (×10^4^ /μL)	20.3 ± 4.6	20.2 ± 4.7	0.45	21.3 ± 6.8	21.8 ± 6.0	0.25
Type IV collagen 7 s	4.61 ± 1.13	4.39 ± 1.06	0.10	5.59 ± 2.51	5.42 ± 1.49	0.13
Glutathione in protein fraction (μM)	1.53 ± 0.92	0.88 ± 0.53	0.004	1.27 ± 0.83	1.00 ± 0.77	0.24
Glutathione in deprotenized fraction (μM)	0.017 ± 0.020	0.019 ± 0.029	0.60	0.036 ± 0.057	0.018 ± 0.017	0.19
CAP (db/m)	300.3 ± 41.1	285.1 ± 53.2	0.049	290.4 ± 50.2	285.8 ± 44.9	0.31
LSM (kPa)	8.71 ± 4.63	7.91 ± 4.22	0.19	11.36 ± 5.05	10.9 ± 4.38	0.32

Data are expressed as mean ± standard deviation

*Abbreviations: BMI* body mass index, *FBS* fasting blood sugar, *IRI* immunoreactive insulin, *HBA1c* hemoglobin A1c, *HDL cholesterol* high density lipoprotein cholesterol, *LDL cholesterol* low density lipoprotein cholesterol, *NEFA* non-esterified fatty acid, *AST* aspartate aminotransferase, *ALT* alanine aminotransferase, *GGT* γ-glutamyl transpeptidase, *CAP* controlled attenuation parameter, *LSM* liver stiffness measurement

## Discussion

Glutathione has a long history for the treatment of chronic liver disease by intravenous injection. This study demonstrates a therapeutic effect of glutathione by oral administration in patients with NAFLD. The primary outcome of this study was a change in ALT levels. The 29 patients who were treated with oral administration of glutathione (300 mg/day) for 4 months showed a reduction in ALT levels as well as reductions in triglycerides, NEFA, and ferritin levels. The findings of the current study suggest the beneficial effects of glutathione by oral administration for NAFLD patients. It is thought that glutathione is degraded into amino acids during digestion and absorption processes. Orally administered glutathione is suggested to serve as a source of amino acids in the synthesis of endogenous glutathione. Supplementation of large doses of glycine and serine, precursors of glutathione, can attenuate NAFLD in human and animal models [18, 19]. In the current study, the dose of glutathione was 300 mg/day. The amount of cysteine potentially released from 300 mg of glutathione is less than 120 mg, the amount that can be obtained from 10 to 20 g of meat or 100 mL of milk. It is, therefore, very unlikely that the current dose of orally administered glutathione attenuates the pathogenesis of NAFLD via an amino acid source for glutathione synthesis.

It is reported that the level of the protein-bound form of glutathione increases 1–2 h after ingestion of glutathione, which suggests that orally administered glutathione is absorbed into the blood [12]. This protein-bound glutathione may be deposited in the liver, attenuating hepatitis.

The levels of protein-bound glutathione were reported to return to baseline levels after an overnight fast [12]. In the current study, we found that the baseline level of the protein-bound form of glutathione significantly decreased after an overnight fast following 4 months of glutathione administration, especially in ALT responders. The levels of protein-bound glutathione in patients in the current study were considerably higher than those of healthy volunteers in previous studies [12] estimated using the same method. Glutathione treatment also decreased protein-bound glutathione to normal baseline levels. These findings suggest that oral administration of glutathione may increase the incorporation of protein-bound glutathione into the liver or decrease the pathological excretion of glutathione from the liver.

NAFLD is a complex disease. Its pathogenesis is thought to involve various factors, including insulin resistance, lipotoxicity, gut/nutrient-derived signals, adipocytokines, oxidative stress, and genetic factors. Dyslipidemia has been reported in 20–80% of patients with NAFLD [20]. Our previously study revealed that orally administrated glutathione accelerates fatty acid utilization by upregulating levels of the protein peroxisome proliferator-activated receptor-γ coactivator-1α and mitochondrial DNA with reduced plasma NEFA levels [21]. The current study also revealed that 24 (82.8%) of our patients had dyslipidemia, and glutathione treatment reduced triglyceride and NEFA levels significantly.

Increases in ferritin and body iron stores have been detected frequently in NAFLD patients [22, 23]. Ferritin and iron can promote the development of NAFLD through oxidative stress [24]. Results from the PIVENS trial showed that oral administration of the anti-oxidant vitamin E improved liver dysfunction and the pathological conditions of NASH [17]. However, long-term treatment with vitamin E has been associated with increases in all-cause mortality and the risk for prostate cancer [25–27], suggesting the need to evaluate the efficacy and safety of this agent. In the current study, glutathione treatment significantly decreased ferritin levels, but the mechanism behind the decrease remains unclear. Glutathione is thought to ameliorate hyperferritinemia and oxidative stress, and to have therapeutic effects in patients with NAFLD.

Liver fat was non-invasively assessed using VCTE with CAP. A meta-analysis found that CAP has good sensitivity and specificity for detecting liver fat [28]. CAP values in our study tended to decrease in all patients and significantly decreased in ALT responders following 4 months of glutathione treatment. Although the relationship between histologic improvement of hepatic steatosis and the reduction of CAP values has not yet been determined, glutathione may reduce hepatic steatosis.

We also investigated the patient factors associated with the therapeutic effects of glutathione. We found that HDL cholesterol and LDL cholesterol levels were higher and HbA1c levels lower in ALT responders than in non-responders. Although the percentage of patients using statins did not differ significantly between the two groups, the percentage tended to be lower in ALT responders than in ALT non-responders. While it can be nothing more than speculation because of the small sample size, patients who showed therapeutic effects following glutathione treatment appeared to be younger and did not have diabetes or had mild diabetes.

Three patients withdrew from the study because of fatigue, elevated blood pressure, and a rash. In ALT responders, HbA1c levels increased and HDL cholesterol levels decreased after glutathione treatment. A study of 6522 patients found that 24 (0.4%) had experienced adverse reactions, the most frequent being anorexia, nausea, vomiting, and rash [29]. Although administration of glutathione may have been associated with a rash in one patient in the current study, the causal associations between glutathione and other adverse effects are unclear.

This study had some limitations. First, our study was a single-arm study without a control group. Second, the study was limited by the small sample size and the short treatment period (4 months). Third, as the pathological conditions of the patients were not evaluated by liver biopsy, incorporation of orally administered glutathione in the liver was not confirmed. Fourth, a number of patients withdrew from the study but no causal association can be determined.

## Conclusions

Treatment with glutathione significantly improved ALT levels. In addition, CAP values were significantly reduced in ALT responders. Our pilot study suggests that oral administration of glutathione supports hepatic metabolism and improves NAFLD. To elucidate the mechanism behind the beneficial effects of glutathione, further studies that examine the incorporation of orally administrated glutathione into the liver and the effects on the host redox system using stable isotope-labeled glutathione and animal models are required. Large-scale clinical trials are necessary to confirm the therapeutic effects of glutathione.

## Abbreviations

ALT: Alanine aminotransferase
AST: Aspartate aminotransferase
BMI: Body mass index
CAP: Controlled attenuation parameter
FBS: Fasting blood sugar
HbA1c: hemoglobin A1c
HDL: High-density lipoprotein
IRI: Immunoreactive insulin
LDL: Low-density lipoprotein
LSM: Liver stiffness measurement
NAFLD: Nonalcoholic fatty liver disease
NASH: Nonalcoholic steatohepatitis
NEFA: Non-esterified fatty acid
UMIN: University Hospital Medical Information Network
VCTE: Vibration-controlled transient elastography
